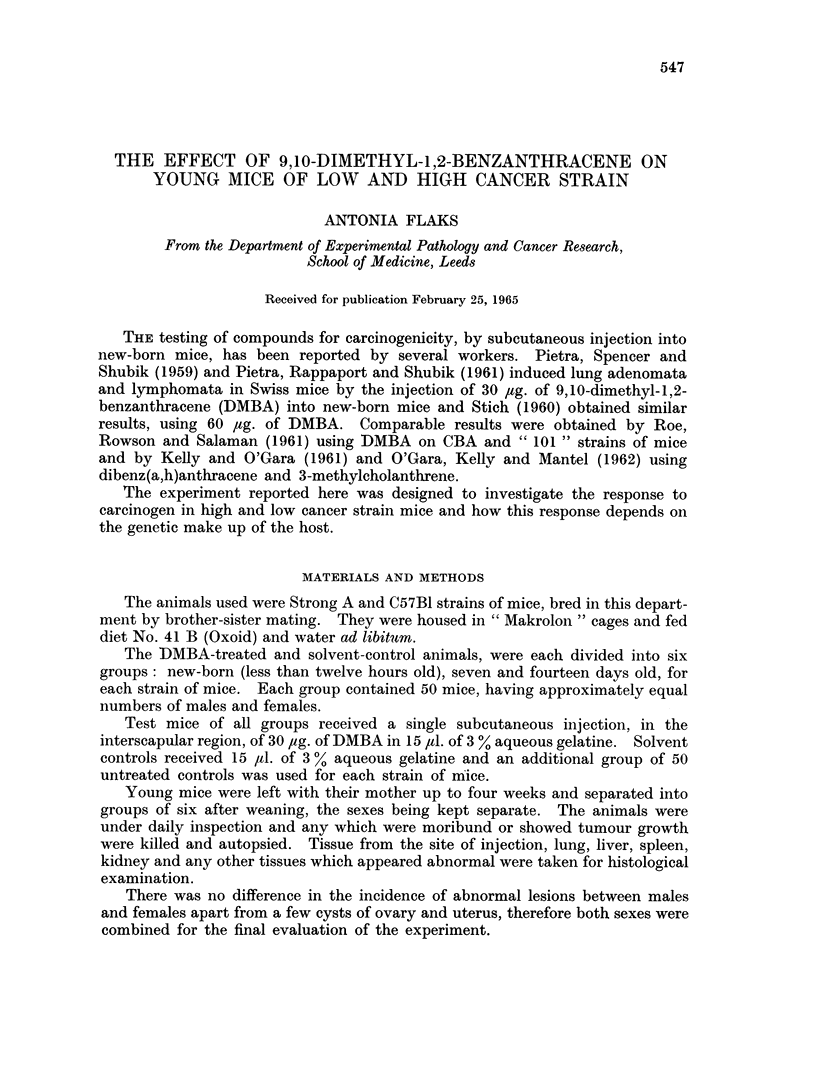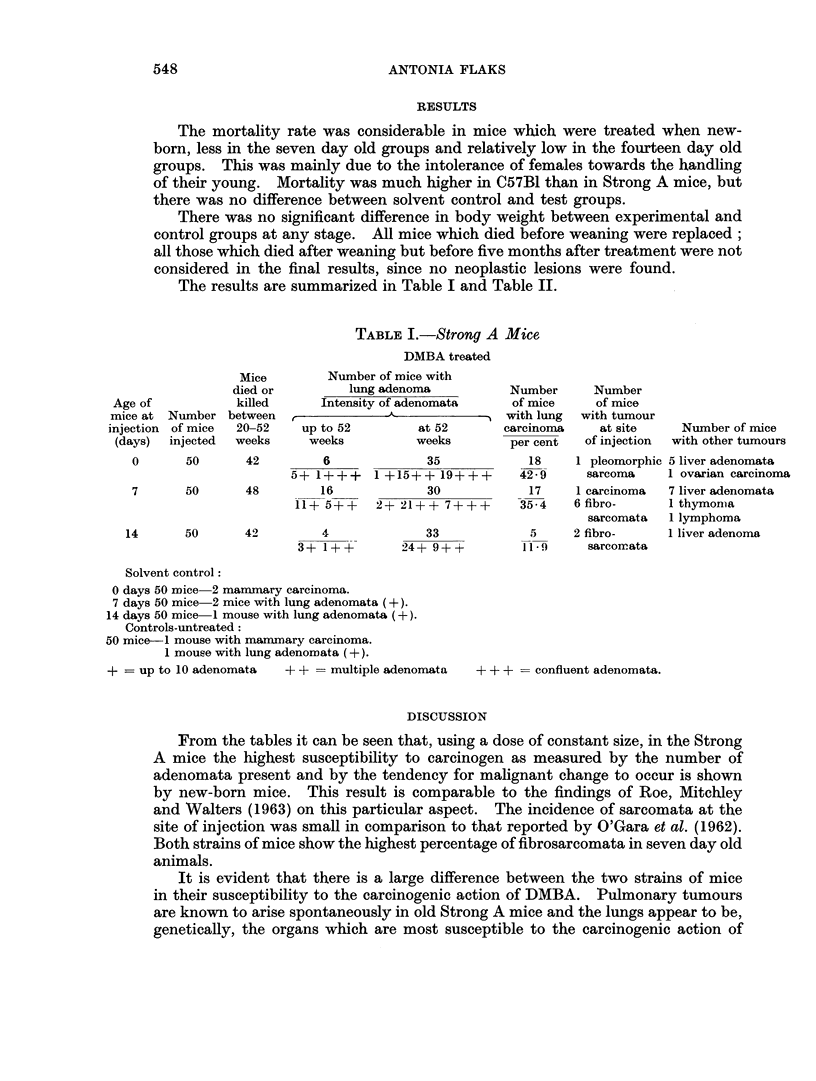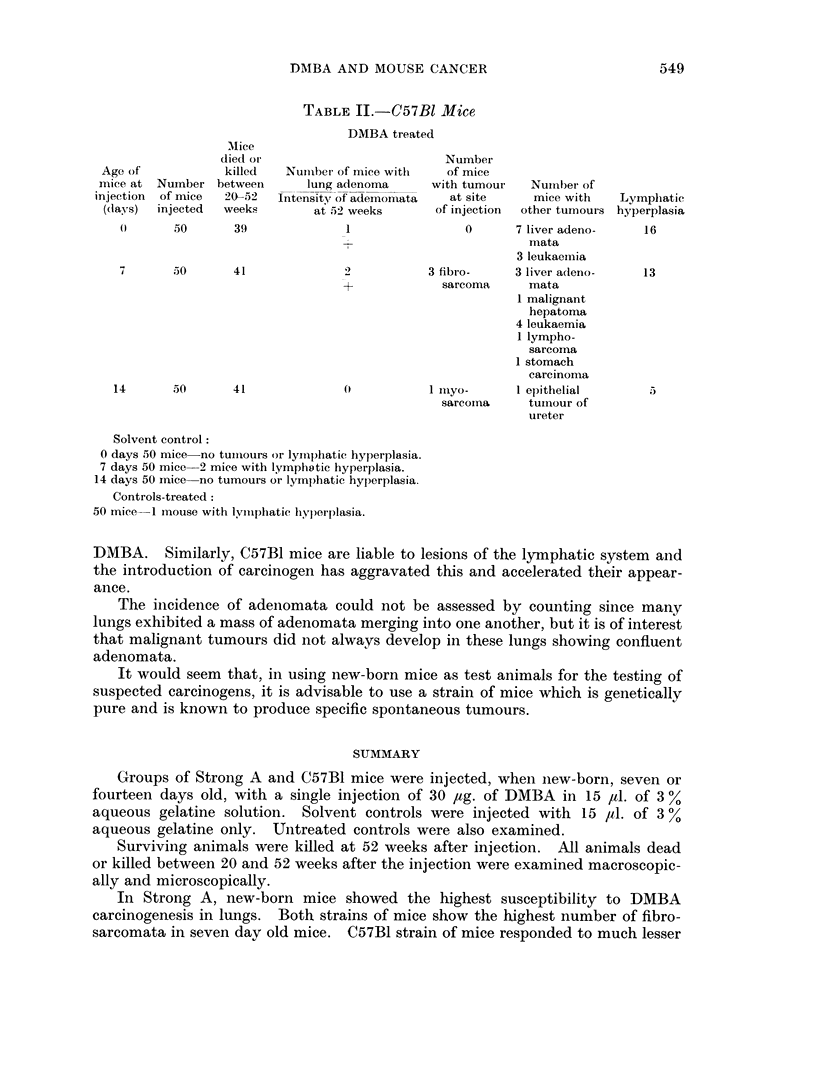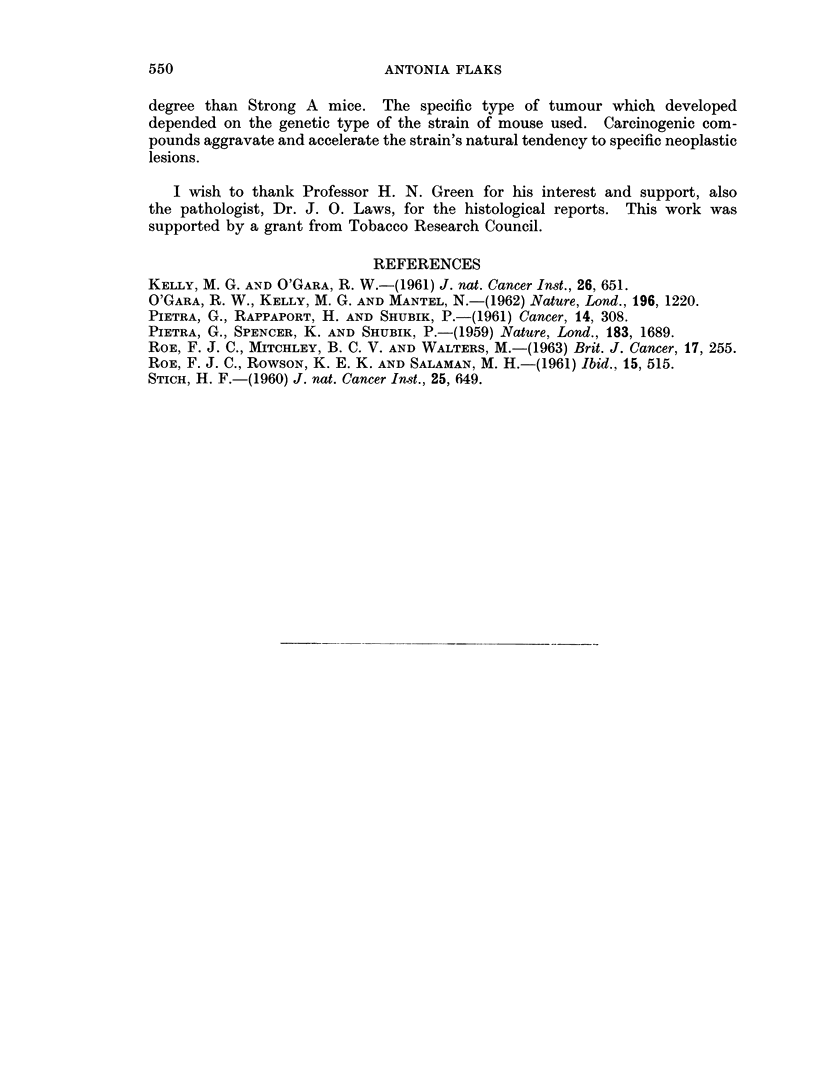# The effect of 9,10-dimethy-1,2-benzanthracene on young mice of low and high cancer strain.

**DOI:** 10.1038/bjc.1965.67

**Published:** 1965-09

**Authors:** A. Flaks


### THE EFFECT OF 9,10-DIMETHYL-1,2-BENZANTHRACENE ON YOUNG MICE OF LOW AND HIGH CANCER STRAIN

ANTONIA FLAKS

*From the Department of Experimental Pathology and Cancer Research, School of Medicine, Leeds*

Received for publication February 25, 1965

THE testing of compounds for carcinogenicity, by subcutaneous injection into new-born mice, has been reported by several workers. Pietra, Spencer and Shubik (1959) and Pietra, Rappaport and Shubik (1961) induced lung adenomata and lymphomata in Swiss mice by the injection of 30 $\mu$g. of 9,10-dimethyl-1,2-benzanthracene (DMBA) into new-born mice and Stich (1960) obtained similar results, using 60 $\mu$g. of DMBA. Comparable results were obtained by Roe, Rowson and Salaman (1961) using DMBA on CBA and " 101 " strains of mice and by Kelly and O'Gara (1961) and O'Gara, Kelly and Mantel (1962) using dibenz(a,h)anthracene and 3-methylcholanthrene.

The experiment reported here was designed to investigate the response to carcinogen in high and low cancer strain mice and how this response depends on the genetic make up of the host.

### MATERIALS AND METHODS

The animals used were Strong A and C57Bl strains of mice, bred in this department by brother-sister mating. They were housed in " Makrolon " cages and fed diet No. 41 B (Oxoid) and water *ad libitum.*

The DMBA-treated and solvent-control animals, were each divided into six groups : new-born (less than twelve hours old), seven and fourteen days old, for each strain of mice. Each group contained 50 mice, having approximately equal numbers of males and females.

Test mice of all groups received a single subcutaneous injection, in the interscapular region, of 30 $\mu$g. of DMBA in 15 $\mu$l. of 3 % aqueous gelatine. Solvent controls received 15 $\mu$l. of 3 % aqueous gelatine and an additional group of 50 untreated controls was used for each strain of mice.

Young mice were left with their mother up to four weeks and separated into groups of six after weaning, the sexes being kept separate. The animals were under daily inspection and any which were moribund or showed tumour growth were killed and autopsied. Tissue from the site of injection, lung, liver, spleen, kidney and any other tissues which appeared abnormal were taken for histological examination.

There was no difference in the incidence of abnormal lesions between males and females apart from a few cysts of ovary and uterus, therefore both sexes were combined for the final evaluation of the experiment.

### RESULTS

The mortality rate was considerable in mice which were treated when new-born, less in the seven day old groups and relatively low in the fourteen day old groups. This was mainly due to the intolerance of females towards the handling of their young. Mortality was much higher in C57Bl than in Strong A mice, but there was no difference between solvent control and test groups.

There was no significant difference in body weight between experimental and control groups at any stage. All mice which died before weaning were replaced ; all those which died after weaning but before five months after treatment were not considered in the final results, since no neoplastic lesions were found.

The results are summarized in Table I and Table II.

TABLE I.—*Strong A Mice*

DMBA treated

| Age of mice at injection (days) | Number of mice injected | Mice died or killed between 20–52 weeks | Number of mice with lung adenoma / Intensity of adenomata: up to 52 weeks | Number of mice with lung adenoma / Intensity of adenomata: at 52 weeks | Number of mice with lung carcinoma / per cent | Number of mice with tumour at site of injection | Number of mice with other tumours |
|---|---|---|---|---|---|---|---|
| 0 | 50 | 42 | 6<br>5+ 1+++ | 35<br>1 +15++ 19+++ | 18<br>42·9 | 1 pleomorphic sarcoma | 5 liver adenomata<br>1 ovarian carcinoma |
| 7 | 50 | 48 | 16<br>11+ 5++ | 30<br>2+ 21++ 7+++ | 17<br>35·4 | 1 carcinoma<br>6 fibro-sarcomata | 7 liver adenomata<br>1 thymoma<br>1 lymphoma |
| 14 | 50 | 42 | 4<br>3+ 1++ | 33<br>24+ 9++ | 5<br>11·9 | 2 fibro-sarcomata | 1 liver adenoma |

Solvent control :
0 days 50 mice—2 mammary carcinoma.
7 days 50 mice—2 mice with lung adenomata (+).
14 days 50 mice—1 mouse with lung adenomata (+).
Controls-untreated :
50 mice—1 mouse with mammary carcinoma.
1 mouse with lung adenomata (+).

+ = up to 10 adenomata ++ = multiple adenomata +++ = confluent adenomata.

### DISCUSSION

From the tables it can be seen that, using a dose of constant size, in the Strong A mice the highest susceptibility to carcinogen as measured by the number of adenomata present and by the tendency for malignant change to occur is shown by new-born mice. This result is comparable to the findings of Roe, Mitchley and Walters (1963) on this particular aspect. The incidence of sarcomata at the site of injection was small in comparison to that reported by O'Gara *et al.* (1962). Both strains of mice show the highest percentage of fibrosarcomata in seven day old animals.

It is evident that there is a large difference between the two strains of mice in their susceptibility to the carcinogenic action of DMBA. Pulmonary tumours are known to arise spontaneously in old Strong A mice and the lungs appear to be, genetically, the organs which are most susceptible to the carcinogenic action of

TABLE II.—*C57Bl Mice*

DMBA treated

| Age of mice at injection (days) | Number of mice injected | Mice died or killed between 20–52 weeks | Number of mice with lung adenoma / Intensity of adenomata at 52 weeks | Number of mice with tumour at site of injection | Number of mice with other tumours | Lymphatic hyperplasia |
|---|---|---|---|---|---|---|
| 0 | 50 | 39 | 1 + | 0 | 7 liver adenomata; 3 leukaemia | 16 |
| 7 | 50 | 41 | 2 + | 3 fibrosarcoma | 3 liver adenomata; 1 malignant hepatoma; 4 leukaemia; 1 lymphosarcoma; 1 stomach carcinoma | 13 |
| 14 | 50 | 41 | 0 | 1 myosarcoma | 1 epithelial tumour of ureter | 5 |

Solvent control :
0 days 50 mice—no tumours or lymphatic hyperplasia.
7 days 50 mice—2 mice with lymphatic hyperplasia.
14 days 50 mice—no tumours or lymphatic hyperplasia.

Controls-treated :
50 mice—1 mouse with lymphatic hyperplasia.

DMBA. Similarly, C57Bl mice are liable to lesions of the lymphatic system and the introduction of carcinogen has aggravated this and accelerated their appearance.

The incidence of adenomata could not be assessed by counting since many lungs exhibited a mass of adenomata merging into one another, but it is of interest that malignant tumours did not always develop in these lungs showing confluent adenomata.

It would seem that, in using new-born mice as test animals for the testing of suspected carcinogens, it is advisable to use a strain of mice which is genetically pure and is known to produce specific spontaneous tumours.

### SUMMARY

Groups of Strong A and C57Bl mice were injected, when new-born, seven or fourteen days old, with a single injection of 30 $\mu$g. of DMBA in 15 $\mu$l. of 3% aqueous gelatine solution. Solvent controls were injected with 15 $\mu$l. of 3% aqueous gelatine only. Untreated controls were also examined.

Surviving animals were killed at 52 weeks after injection. All animals dead or killed between 20 and 52 weeks after the injection were examined macroscopically and microscopically.

In Strong A, new-born mice showed the highest susceptibility to DMBA carcinogenesis in lungs. Both strains of mice show the highest number of fibrosarcomata in seven day old mice. C57Bl strain of mice responded to much lesser degree than Strong A mice. The specific type of tumour which developed depended on the genetic type of the strain of mouse used. Carcinogenic compounds aggravate and accelerate the strain's natural tendency to specific neoplastic lesions.

I wish to thank Professor H. N. Green for his interest and support, also the pathologist, Dr. J. O. Laws, for the histological reports. This work was supported by a grant from Tobacco Research Council.

### REFERENCES

KELLY, M. G. AND O'GARA, R. W.—(1961) *J. nat. Cancer Inst.*, **26**, 651.
O'GARA, R. W., KELLY, M. G. AND MANTEL, N.—(1962) *Nature, Lond.*, **196**, 1220.
PIETRA, G., RAPPAPORT, H. AND SHUBIK, P.—(1961) *Cancer*, **14**, 308.
PIETRA, G., SPENCER, K. AND SHUBIK, P.—(1959) *Nature, Lond.*, **183**, 1689.
ROE, F. J. C., MITCHLEY, B. C. V. AND WALTERS, M.—(1963) *Brit. J. Cancer*, **17**, 255.
ROE, F. J. C., ROWSON, K. E. K. AND SALAMAN, M. H.—(1961) *Ibid.*, **15**, 515.
STICH, H. F.—(1960) *J. nat. Cancer Inst.*, **25**, 649.